# Effects of water ammonia nitrogen on hemolymph and intestinal microbiota of *Litopenaeus vannamei*

**DOI:** 10.1007/s44307-023-00008-2

**Published:** 2024-01-26

**Authors:** Xuanting Li, Xisha Deng, Dongwei Hou, Shenzheng Zeng, Zhixuan Deng, Renjun Zhou, Lingyu Zhang, Qilu Hou, Qi Chen, Shaoping Weng, Jianguo He, Zhijian Huang

**Affiliations:** 1 State Key Laboratory of Biocontrol, Southern Marine Sciences and Engineering Guangdong Laboratory (Zhuhai), School of Marine Sciences, Sun Yat-Sen University, Guangzhou, 510275 P. R. China; 2 School of Life Sciences, Sun Yat-Sen University, Guangzhou, 510275 P. R. China; 3 Maoming Branch, Guangdong Laboratory for Lingnan Modern Agricultural Science and Technology, Maoming, 525435 P. R. China

**Keywords:** *Litopenaeus vannamei*, Ammonia nitrogen, Hemolymph, Hepatopancreas, Intestinal microbiota

## Abstract

Ammonia stress was detrimental to shrimp, but how water ammonia nitrogen (ammonia-N) influences the shrimp’s health remains unclear. Thus, this study was designed to investigate the effects of water ammonia-N on hemolymph ammonia-N concentration, hepatopancreas structure, and the intestinal microbiota of *Litopenaeus vannamei* with four experiments. We found that the concentration of ammonia-N in shrimp hemolymph was significantly higher than that in pond water, indicating that water ammonia-N stimulates the accumulation of hemolymph ammonia-N. Results also indicated that the hemolymph ammonia-N accumulation would disrupt the hepatopancreas structure and alter the intestinal microbial composition. The concentration of hemolymph ammonia-N and severity of hepatopancreas damage positively correlated with water ammonia-N concentration. However, though the diversity of intestinal microbiota was varied by ammonia-N, there were no significant differences between groups, suggesting that the variation was relatively minimal. Furthermore, returning shrimp to pristine water after ammonia-N exposure could reduce the hemolymph ammonia-N concentration and the mortality rate. This study provides evidence of temporal variations in hemolymph ammonia-N concentration, hepatopancreatic structure, and intestinal microbiota under different water ammonia-N levels, which might shed insights into ecological cognition on scientific management of shrimp culture and microecological prevention of shrimp health.

**Supplementary Information:**

The online version contains supplementary material available at 10.1007/s44307-023-00008-2.

## Introduction

Aquatic foods, also referred to as “Blue Foods,” captured from or cultivated in water ecosystems, have attracted significant attention around the world for their high-quality protein and valuable nutrients (Naylor et al. 2021). The Pacific white shrimp (*Litopenaeus vannamei, L. vannamei*) is an increasingly important aquatic economic species in the aquaculture industry. Given that growing consumption of seafood requires higher yields in aquatic products, intensive cultivation becomes the major supply of many crucial crustacean species (Food and Agriculture Organization of the United Nations, 2020). However, intensive cultivation not only aggravates the accumulation of waste products, but also the decomposition of feces and baits, which ultimately result in water deterioration and a surge of aquacultural diseases. Shrimp diseases, such as white feces syndrome (WFS), acute hepatopancreatic necrosis disease (AHPND), and hepatopancreas necrosis syndrome (HPNS), tremendously strike global shrimp production (Dongwei Hou et al. 2018a; Huang et al. 2020, 2016; Lee et al. 2015). In particular, ammonia nitrogen (ammonia-N) easily accumulates in the process of shrimp culture and has a strong toxic effect on the survival, physiological metabolism, and immune function of cultured shrimp (Chang et al. 2015; Romano and Zeng 2013).

Ammonia-N, presenting as NH_3_ and NH_4_^+^, is a common end product of protein and amino acid metabolism and also the most common contaminant in aquaculture ecosystems (Regnault 1987; Weihrauch and Allen 2018; Zimmer et al. 2017). Though proven to involve multiple metabolic processes, environmental ammonia-N has deleterious effects on aquatic animals, especially at high concentrations, which are prominent in disorders of metabolism, suppression of immune functions, and alternation in neuroendocrine control (Lv et al. 2021; Si et al. 2019; Wang et al. 2021). In the circulatory system of aquatic animals, ammonia-N accumulation directly interrupts coagulation, alters the concentration of hemolymph protein and amino acid, and even leads to high mortality, which points out the importance of removing ammonia-N from the internal environment (Chang et al. 2015; Chen and Lei 1990; Lin and Chen 2001). Due to the diversity of habitats and varied sensitivity to ammonia-N, strategies for maintaining ammonia homeostasis differ dramatically between aquatic animals. Some aquatic animals deploy ammonia detoxification by transforming ammonia into less toxic compounds like urea and uric acid (Weihrauch and Allen 2018). Others, such as *Palaemonetes varians* and *Crangon crangon*, in which ammonia accounts for around 95% of total excreted nitrogen, primarily rely on ammonia excretion (Regnault and Lagardere 1983; Williams and Snow 1971). Therefore, it is crucial to explore the mechanisms of ammonia-N regulation for different aquatic species.

The physical and chemical parameters (pH, temperature, salinity, dissolved oxygen, ammonia, and so on) of pond water have been proven to strongly impact animal health in previous studies (Tovar et al., 2000; Visscher and Duerr 1991). Ammonia, nitrite, and nitrate are the most common forms of inorganic nitrogen in ponds and also important parameters for water quality evaluation (Valencia-Castañeda et al. 2019). A study in industrial aquaculture ponds demonstrates that the concentration of ammonia-N fluctuated drastically during the *L. vannamei* culture period and became one of the major stressors of shrimp health (Liu et al. 2017). It is reported that shrimp exposed to high environmental ammonia levels showed hemolymph ammonia-N accumulation, hepatopancreas and intestinal mucosal damage, immune and metabolic disruption, and also a shift in the intestinal microbiota, which might interrupt metabolic and immune function and enhance the susceptibility to pathogens (Duan et al. 2018, 2021; Lv et al. 2021; Wang et al. 2021; Weihrauch and Allen 2018; Zhang et al. 2021). There is evidence that elevated ammonia-N in water increases the susceptibility to pathogens in *Oncorhynchus mykiss* and *L. vannamei*, but the underlying mechanisms are still obscure (Hurvitz et al. 1997; Liu et al. 2017). Although several studies have revealed the alteration of intestinal microbial composition in *L. vannamei* when exposed to high concentrations of ammonia-N, the concentration of ammonia-N applied in these studies (15–20 mg·L^−1^) might be much higher than in aquaculture systems (Duan et al. 2018; Duan et al. 2021; Lv et al. 2021). There are also some contradictions in the results. Lv (2021) found that the variation in intestinal microbiota abundance and diversity was unremarkable when shrimp were exposed to an ambient ammonia concentration of 20 mg·L^−1^ for 96 h, while Duan (2021) suggested that the variation was obvious when exposed to 15 mg·L^−1^ for 72 h. Therefore, there is a scarcity of studies to comprehensively examine the alteration in shrimp physiology and intestinal microbiota under multiple low ammonia-N concentration settings (0–10 mg·L^−1^). Besides, since Romano and Zeng showed that the detrimental effects of ammonia-N could be removed by transferring *Portunus pelagicus* to pristine water, further investigation is necessary to determine whether the ammonia-N damage to shrimp could be reparable.

In this study, in an attempt to fully explore the effects of water ammonia-N on *L. vannamei*, water and shrimp hemolymph samples were collected from industrial aquacultural ponds (IAPs) and earthen ponds (EPs) to evaluate the concentration of ammonia-N, nitrite-N, and nitrate–N in shrimp hemolymph and the external water environment. According to the ammonia-N concentration in aquaculture ecosystems, four delicate experiments were designed to thoroughly examine the variation in concentration of hemolymph ammonia-N and ammonia-N excretion rate when exposed to altered water ammonia-N levels. Furthermore, histopathology and 16S rRNA gene amplicon sequencing were combined to characterize hepatopancreas tissue structure and intestinal microbial composition under different concentrations of water ammonia-N. These concomitant variations not only reveal possible links among the hemolymph ammonia-N level, hepatopancreas cells, and intestinal microbiota but also provide a theoretical basis for understanding the mechanisms of ammonia-N regulation in *L. vannamei* under different exposure times and concentrations of water ammonia-N, which may improve our ecological cognition on the scientific management of shrimp culture and the microecological prevention of shrimp health.

## Materials and methods

### Measurement of ammonia-N, nitrite-N, and nitrate-N in water and shrimp hemolymph

A total of 147 shrimp samples and 36 water samples from 12 industrial aquaculture ponds (IAPs), together with 127 shrimp samples and 18 water samples from six earthen ponds (EPs), were collected in Zhuhai, Maoming, Dongfang, and Tianjin cities in China. Hemolymph samples were obtained and preserved according to Yeh and Chen (2009). From the base of the fifth pair of pleopods of shrimp, hemolymph was extracted by a 1.0 mL syringe and thoroughly mixed at a volume ratio of 1:9 with anticoagulant. Water samples taken from the surface of ponds were filtered through 0.22 μm Millex-GP filters with a polyvinylidene fluoride (PVDF) membrane (MerckMillipore, USA) to remove particles, and then collected into 50 mL sterile centrifuge tubes (Yang 2004). All samples were preserved at 4 ℃ until measurement.

To measure the concentrations of hemolymph ammonia-N, nitrite-N, and nitrate-N, hemolymph samples were centrifugalized (4℃, 800 × g, 10 min) to isolate the supernatant (Yeh and Chen 2009). Hemolymph ammonia-N was tested by an ammonia assay kit (Nanjing Jiancheng, China), while nitrite-N and nitrate–N were tested by methods according to Yang (2004). For nitrite-N measurement, a nitrite-N standard curve was prepared with a standard nitrite solution. Then, a certain volume of the supernatant was transferred into a 25 mL standard tube and dilute with water to 25 mL. Before examination, 0.5 mL of sulfanilamide and 0.5 mL of N-(1-naphthyl)-ethylenediamine dihydrochloride were added and mixed thoroughly. Solution absorbance at 540 nm wavelength was measured using a spectrometer and was translated into nitrite-N concentration via the standard curve. The following equation was utilized to calculate the nitrite-N concentration of hemolymph samples.

$$C = {C}_1\times {V}_{1} / {V}_{0}$$

where *C* is the nitrite-N concentration of the hemolymph sample (mg·L^−1^), *C*_1_ is the nitrite-N concentration of the solution being tested, and *V*_1_ is the volume of the solution being tested, and *V*_0_ is the volume of the supernatant added to the standard tube. For nitrate–N measurement, except that imidazole buffer solution should be added into the supernatant samples before samples were filled into standard tubes, subsequent processes were identical to nitrite-N measurement.

The concentrations of water ammonia-N, nitrite-N, and nitrate–N were measured using an automatic discrete analyzer, CleverChem380 (DeChem-Tech, Germany) according to Specification for Oceanography Survey (General Administration of Quality Supervision, Inspection and Quarantine of the People’s Republic of China, 2007; Edgar et al. 2011).

### The ammonia-N concentration of shrimp hemolymph under the different water ammonia-N concentrations

Experimental shrimps with an average weight of 14.08 ± 2.25 g (mean weight ± standard deviation) were from shrimp cultural ponds in Zhuhai, Guangdong, China and placed in a 210 L aquarium (70 cm × 55 cm × 55 cm) contained ammonia-free water (temperature: 27℃, salinity: 5‰, DO ≥ 7 mg·L^−1^, and pH = 7.8) for acclimation. Feeding would be terminated at 24 h (h) before experiments, and no feed would be applied during experiment periods.

Four delicate experiments (named Experiment 1–4) were designed to detect the ammonia-N concentration of shrimp hemolymph under different ammonia-N concentrations in environmental water (Table 1; Supplementary Fig. S1). According to the results of water and hemolymph ammonia-N concentration in ponds [Water: (IAP: 0.63 ± 0.32, EP: 0.22 ± 0.10); Hemolymph: (IAP: 8.99 ± 6.00, EP: 6.43 ± 4.28)], six concentrations of 0 mg·L^−1^, 1 mg·L^−1^, 2.5 mg·L^−1^, 5 mg·L^−1^, 7.5 mg·L^−1^ and 10 mg·L^−1^ were chosen for Experiment 1. And four concentrations of 0 mg·L^−1^, 2.5 mg·L^−1^, 10 mg·L^−1^ and 50 mg·L^−1^ were chosen for Experiment 2 according to the 24 h LC_50_ (59.72 mg·L^−1^ at 15‰, 66.38 mg·L^−1^ at 25‰) described by Lin and Chen (2001).

**Table 1:** Experiment design

Experiment	Number of individual per aquarium	Aquarium volume (L) / (length × width × height) (cm^3^)	Replicates (aquariums)
1	50	80 / (70 × 45 × 80)	A1: 6
			B2.5: 6
			C5: 6
			D7.5: 6
			E10: 6
			Control: 6
2	50	100 / (100 × 60 × 80)	a2.5: 2
			b10: 2
			c50: 2
			c: 2
3	30	30 / (40 × 55 × 65)	E^a^: 3 (AE1, AE2, AE3)
			C^b^: 3(AEC1, AEC2, AEC3)
4	1	5 / (13 × 13 × 25)	E: 45 (ae1-ae45)
			C: 5 (aec1-aec5)

^a^*E* Experiment groups

^b^*C* Control group

#### Experiment 1

After acclimation, shrimps were cultured in different groups with water ammonia-N concentrations of 0 mg·L^−1^ (designated as Control), 1 mg·L^−1^ (designated as A1), 2.5 mg·L^−1^ (designated as B2.5), 5 mg·L^−1^ (designated as C5), 7.5 mg·L^−1^ (designated as D7.5), and 10 mg·L^−1^ (designated as E10). Six tanks were applied for each group. Sampling was carried out at 0 h, 6 h, 12 h, 24 h, 36 h, 48 h, and 72 h. At each sampling point, three shrimp for histopathological analysis and 16S rRNA gene sequencing and six shrimp for hemolymph ammonia-N concentration measurement were obtained from each group. The dissection and preservation of the hepatopancreas and intestines were processed immediately after sample collection according to the methods described in Huang et al. (2020). Shrimps were dissected on ice after surface sterilization using 75% ethanol and 0.85% saline solution. Hepatopancreas and intestines were aseptically isolated and collected in 1.5 mL centrifuge tubes. For histopathological examination, hepatopancreas was soaked in 1 mL Davidson’s fixative at 4℃, and transferred into 50% ethanol after 24 h, and then stored at 4℃ until examination. Intestines were preserved with DNA preservative solution and stored at -80℃ until DNA extraction.

#### Experiment 2

Then, to determine whether shrimps exposed to different environmental ammonia-N concentrations would recover when transferred to ammonia-free water, shrimp were reared in four groups with water ammonia concentrations of 0 mg·L^−1^ (designated as c), 2.5 mg·L^−1^ (designated as a2.5), 10 mg·L^−1^ (designated as b10), and 50 mg·L^−1^ (designated as c50). After 24 h, shrimp in one of the two replicates of each group were transferred to aquariums containing ammonia-free water. The three new groups that received shrimp from c, a2.5, b10 and c50 were designated as Tc, Ta2.5, Tb10, and Tc50 corresponding to the initial groups. During the experiment period, sampling was carried out at 0 h, 6 h, 12 h, 24 h, 27 h, 30 h, 36 h, 48 h, 60 h, 72 h, and 84 h. Four shrimps were collected from each aquarium at each sampling point.

#### Experiment 3

Furthermore, to reveal ammonia accumulation in shrimp hemolymph and its potential effect on ammonia excretion, three aquariums with shrimp reared in ammonia-free water (*n* = 30 per aquarium, designated as AE1, AE2, and AE3) and also three control groups (designated as AEC1, AEC2 and AEC3) without shrimp were set up. At 0 h, 6 h, 12 h, 24 h, 36 h, 48 h, and 72 h, five shrimp and 5 mL of water for ammonia-N concentration measurement were collected from each aquarium.

#### Experiment 4

Forty-five replicates (*n* = 1 per aquarium, designated as ae1-ae45) with just one shrimp reared in each aquarium with ammonia-free water and five control groups (designated as aec1-aec5) without shrimps were set up to measure the ammonia excretion rate of individual shrimps. At 0 h, 6 h, 12 h, 24 h, 36 h, 48 h, 72 h, 96 h, 120 h, and 144 h, 5 mL of water for ammonia-N concentration measurement were collected from each aquarium.

The ammonia-N concentration of water and hemolymph samples were measured as previously described. The ammonia-N excretion rate represents the amount of excreted ammonia-N per hour per gram. Water ammonia concentration data was applied to the following equation to calculate shrimp ammonia excretion rate (*R*, mg-N·g^−1^·h^−1^) according to Jiang et al. (2000):

$$R= [(C_f-C_i ) \times V] / (W \times t)$$

where *C*_f_ is the corrected final concentration of sample (μg-N·L^−1^),* C*_i_ is the initial concentration of sample (μg-N·L^−1^), *V* is the water volume of the tank (L), *W* is the live weight of shrimp (g), and* t* is the time interval between initial and final water sampling (h).

### Histopathological examination

For hematoxylin-eosin staining (H&E staining), hepatopancreas need to be processed for 5 mm paraffin embedding and hematoxylin-eosin staining within a week after sampling. The paraffin-embedded hepatopancreas tissues were cut off at their maximum cross sections, dewaxed in xylene and ethanol, and then stained with a H&E dye solution set (G1003, Servicebio, Wuhan, China). The tissue sections were dehydrated in gradient alcohol, sealed with neutral gum, and examined by light microscopy.

### DNA extraction, PCR amplification and 16S rRNA gene amplicon sequencing

Genomic DNA extraction of intestine samples was carried out using the QIAamp PowerFecal DNA Kit (QIAGEN, USA) following the manufacturer’s instructions. The concentration and purity of DNA samples were determined by a NanoVuePlus spectrophotometer (GE Healthcare, USA) and 1% agarose gels. The universal primer pair 338F (5’-ACTCCTACGGGAGGCAGCAG-3’) and 806R (5’-GGACTACHVGGGTWTCTAAT-3’) was employed to amplify the V3-V4 regions of 16S rRNA gene PCR products from the intestine DNA samples. PCR was performed in 20 µL reactions, with each containing 10 ng of purified DNA as templates, and the following thermocycling conditions were used: pre-denaturation at 95℃ for 30 s, 27 cycles of denaturation at 95℃ for 30 s, annealing at 55℃ for 30 s, and extension at 72℃ for 30 s, with a final elongation at 72℃ for 10 min. The PCR products were purified using 1% agarose gels electrophoresis and AxyPrep DNA Gel Extraction Kit (Axygen Biosciences, Union City, USA), and finally quantified by QuantiFluorTM-ST (Promega, USA). Equimolar amounts of amplicons from each sample were pooled and then sequenced using the Illumina Miseq PE 300 platform (Illumina, San Diego, USA) by Majorbio Bio-Pharm Technology Co Ltd. (Shanghai, China).

Paired-end sequences were merged by FLASH (Magoč and Salzberg 2011) and then processed with Quantitative Insights Into the Microbial Ecology pipeline (QIIME version 1.9.0) (Caporaso et al. 2010). Sequences with ambiguous bases or truncation of more than three consecutive bases with a Phred quality score Q < 20 were deleted. The sequence data have been deposited in the GenBank Sequence Read Archive database. The accession number is PRJNA877447. Bacterial phylotypes were identified via UCLUST (Edgar 2010) and classified into operational taxonomic units (OTUs) at a 97% cutoff, while chimeric sequences were discarded using the UCHIME algorithm (Edgar et al. 2011). Within each OTU, the most abundant sequence was selected as a representative, and its taxonomy was assigned to a closed reference genome by the RDP Classifier algorithm (Silva SSU database 128, Version 2.2, http://rdpcmemsuedu/) with 80% confidence. Alpha diversity, including Shannon, Simpson, and Chao1 indexes, was calculated using QIIME.

### Statistical analysis

Ammonia, nitrite, and nitrate concentration and shrimp ammonia excretion rate are presented as Mean ± SD. Statistical analysis was conducted by SPSS (version 21.0) to evaluate the differences between groups. The Kolmogorov-Smirnov test was employed to determine the normal distribution of data. If the data basically conforms to a normal distribution, Student's *t* test would be applied to evaluate significance between two groups, and the analysis of variance (ANOVA) with the Tuckey test would be applied to evaluate significance among multiple experimental groups. Otherwise, the Mann-Whitney U test would be applied when the data was not normally distributed. To analyze the similarity of bacterial composition among groups, non-metric multidimensional scaling (NMDS) analysis was performed based on the Bray-Curtis distance. The significance was determined using permutational multivariate analysis of variance (PERMANOVA).

## Results

### Comparison of ammonia-N, nitrite-N, and nitrate–N concentration between water and hemolymph in shrimp culture ecosystems

**Fig. 1:**
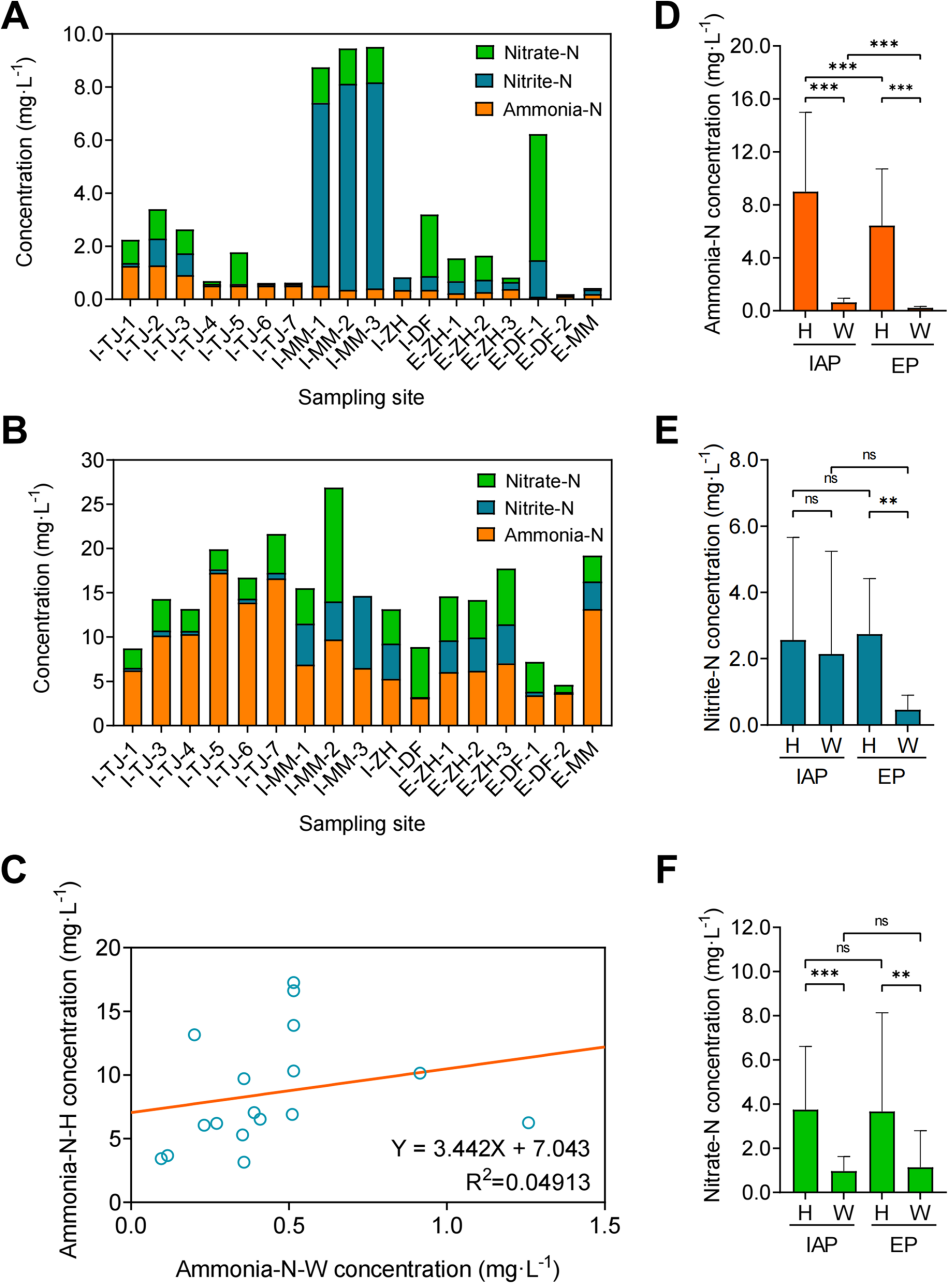
Comparison of concentration of ammonia-N, nitrite-N, and nitrate-N in water and shrimp hemolymph among different aquaculture systems. **A** Average water ammonia-N, nitrite-N, and nitrate-N concentration in different sites. **B** Average shrimp hemolymph ammonia-N, nitrite-N, and nitrate-N concentration in different sites. I: Industrial aquaculture pond; E: Earthen pond. TJ: Tianjin; MM: Maoming; ZH: Zhuhai; DF: Dongfang. **C** Correlation between concentration of water ammonia-N and hemolymph ammonia-N determined by linear regression analysis. H: Hemolymph; W: Water. **D**-**F** Comparison of ammonia-N (**D**), nitrite-N (**E**), and nitrate-N (**F**) concentration in shrimp hemolymph and water of industrial aquaculture ponds (*n* = 12) and earthen ponds (*n* = 6). Each bar represents the Mean ± SD value from sarmples of industrial aquaculture ponds or earthen ponds. Significant difference was determined using Student’s *t* test and indicated by asterisks (ns, no significance; *, *P* < 0.05; **, *P* < 0.01; ***, *P* < 0.005)

The average concentration of water and hemolymph ammonia-N, nitrite-N, and nitrate-N from IAPs and EPs were shown in Fig. 1A and B. Ammonia-N composed almost the largest part of hemolymph non-organic N component across sampling sites. The concentration of water and hemolymph ammonia-N, nitrite-N, and nitrate-N fluctuated drastically among sampling sites and did not show any significant correlation between each other (Fig. 1A and B; Supplementary Table S1). Comparison between IAPs and EPs showed that ammonia-N, nitrite-N, and nitrate-N were enriched in shrimp hemolymph, which were respectively 13.3, 1.88, and 2.49 folds higher than in the surrounding water of IAP, while 23.3, 11.6, and 8.7 folds higher than in the surrounding water of EP (Supplementary Table S2). As for pond water, compared with EPs (0.10 mg·L^−1^ -0.39 mg·L^−1^), IAPs (0.35 mg·L^−1^ -1.28 mg·L^−1^) had a significantly higher average concentration of ammonia-N (*P* < 0.005, Mann-Whitney U test). Such a difference in pond water might be due to the relative high stocking density of the IAPs in this study. Meanwhile, the concentration of hemolymph ammonia-N was also significantly higher in IAPs (*P* < 0.005, Mann–Whitney U test), while neither the concentration of nitrite-N nor nitrate–N showed significant differences between IAPs and EPs (*P* = 0.5233, *P* = 0.1401, Mann–Whitney U test) (Fig. 1D, E and F). Linear regression analysis indicated a positive tendency between concentration of water ammonia-N and shrimp hemolymph at various sampling sites (Fig. 1C). Thus, these data demonstrated that, when compared to pond water, the concentration of ammonia-N was higher in IAPs and was relatively enriched in shrimp hemolymph. Additionally, there might be a positive correlation between the ammonia-N concentration in shrimp hemolymph and the ammonia-N concentration in water.

### Variation in concentration of shrimp hemolymph ammonia-N when exposed to different concentrations of water ammonia-N

**Fig. 2:**
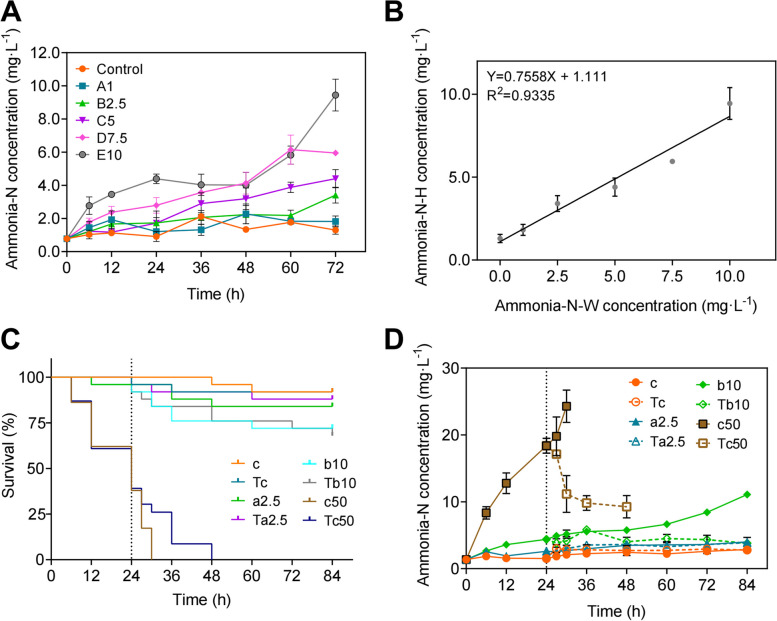
Accumulation of ammonia-N in shrimp hemolymph when exposed to different water ammonia-N concentration. **A** Shrimp hemolymph ammonia-N concentration in Experiment 1. **B** Correlation between hemolymph and water ammonia-N concentration at 72 h in Experiment 1 determined by linear regression analysis. **C** Survival curve of different groups in Experiment 2. **D** Shrimp hemolymph ammonia-N concentration in Experiment 2

In experiment 1, to reveal the relationship between the concentration of water ammonia-N and shrimp hemolymph ammonia-N, shrimp were divided into six groups and exposed to different water ammonia-N concentrations. Time-dependent alteration of ammonia-N concentration in hemolymph was presented in Fig. 2A, which showed a significant elevation of ammonia-N concentration in B2.5, C5, D7.5, and E10 when these groups were compared with group Control (*P* < 0.05, *P* < 0.05, *P* < 0.005,* P* < 0.005, ANOVA) (Supplementary Table S3). In group Control, A1, B2.5, C5, D7.5, and E10, the average concentrations of hemolymph ammonia-N at 72 h were 1.3, 1.82, 3.41, 4.4, 5.95, and 9.44 mg·L^−1^ respectively, with significant differences among A1, B2.5, C5, D7.5, and E10 (*P* < 0.05, Tukey’s HSD comparison test) (Fig. 2A; Supplementary Tables S3 and S4). Although no significant difference was detected between Control and A1, a higher concentration of hemolymph ammonia-N was observed in A1 at 72 h (Supplementary Tables S3 and S4). Since the concentration of hemolymph ammonia-N at 0 h was at the same level among groups, linear regression analysis showed that the concentrations of hemolymph ammonia-N at 72 h of different groups were positively correlated with the concentrations of water ammonia-N (Fig. 2B), which indicated that ammonia-N accumulates in shrimp hemolymph when exposed to environmental ammonia-N, and the increase in water ammonia-N concentration and exposure time might accelerate ammonia-N accumulation in hemolymph.

To investigate whether the effects on the concentration of hemolymph ammonia-N persist when the concentration of water ammonia-N alters, Experiment 2 was carried out by transferring shrimp to ammonia-free water after being exposed to different concentrations of water ammonia-N. The mortality rate in c50 and Tc50 reached 100% at 30 h and 48 h, while in c, Tc, a2.5, Ta2.5, b10, and Tb10 were 4%, 4%, 8%, 6%, 14%, and 16%, respectively (Fig. 2C; Supplementary Table S5), which indicated that ammonia-N in surrounding water might pose a negative effect on shrimp health status. Besides, the concentration of hemolymph ammonia-N increased in group b10 and c50 but decreased in Tb10 and Tc50 after transfer (*P* < 0.005, *P* < 0.05, ANOVA) (Fig. 2D; Supplementary Table S6). No significant difference was observed in the concentration of hemolymph ammonia-N when Tc and Ta2.5 were compared with the corresponding untreated groups (c and a2.5) (*P* = 0.9994, *P* > 0.9999, ANOVA) (Supplementary Tables S6 and S7). The concentration of hemolymph ammonia-N at 84 h is presented in Supplementary Table S6, which indicates a positive trend between the concentration of water and hemolymph ammonia-N in both the transfer groups and the corresponding untreated groups. These results showed that the concentration of hemolymph ammonia-N and mortality were dramatically reduced after environmental ammonia-N was eliminated, especially in groups exposed to higher water ammonia-N concentrations. However, given that the mortality in Tc50 reached 100% at 30 h, it is possible that the negative effects brought by water ammonia-N might become irreversible when the concentration of water ammonia-N exceeds a certain threshold.

**Fig. 3:**
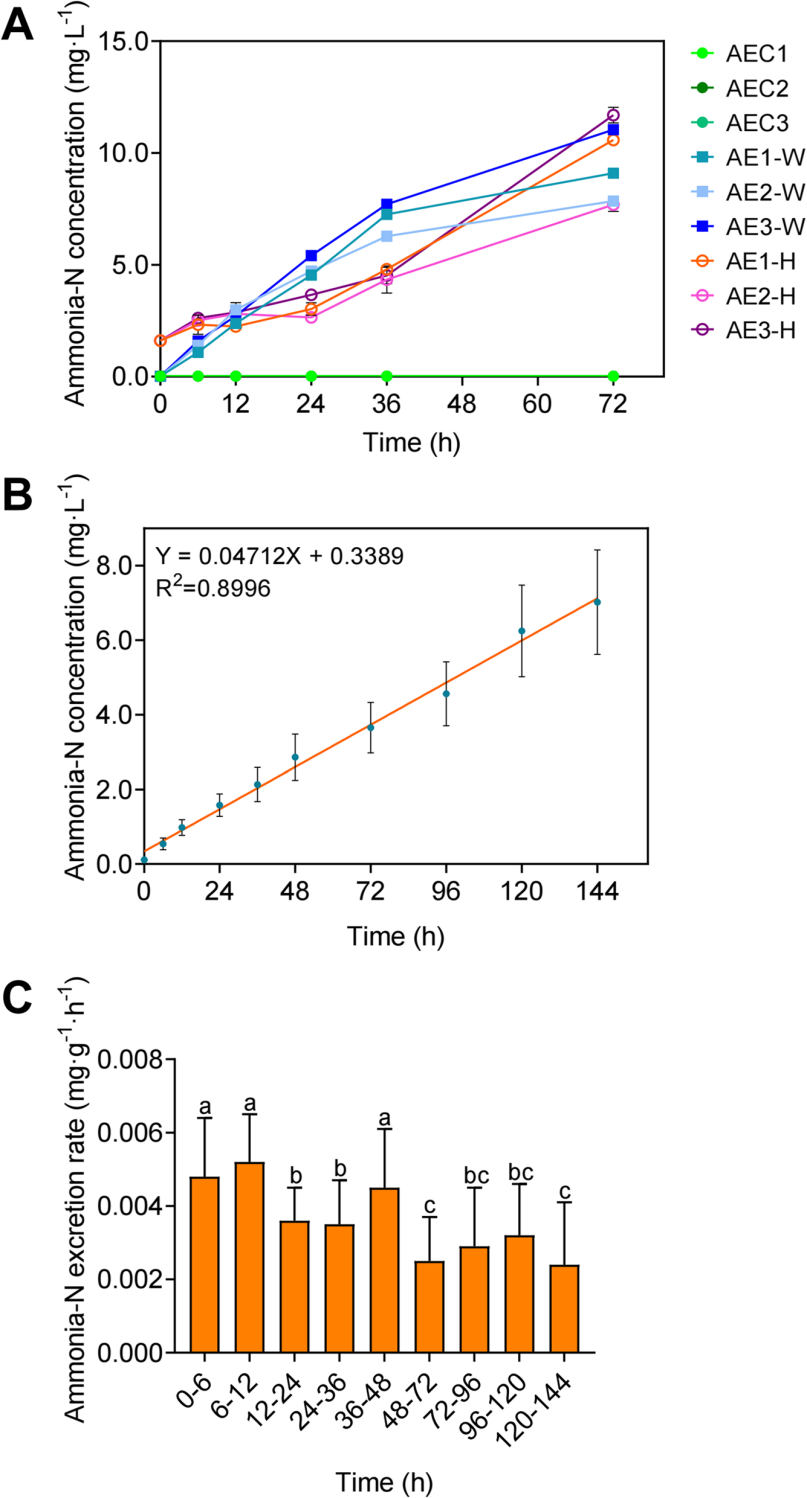
Alteration in shrimp ammonia-N excretion in ammonia-N free water. **A** Shrimp hemolymph and water ammonia-N concentration in Experiment 3. **B** Correlation between ammonia-N concentration and rearing time determined by linear regression analysis. **C** Ammonia-N excretion rate in Experiment 4. Different letters (**a**, **b** and **c**) denote a statistically significant difference using a Tukey’s HSD comparison test (*P* < 0.05)

In consideration of the potential negative effects on shrimp health status and the accumulating hemolymph ammonia-N over time, we hypothesized that water ammonia-N might inhibit shrimp ammonia-N excretion. Experiment 3 and 4 were subsequently conducted to verify this hypothesis by estimating the shrimp ammonia-N excretion rate in an ammonia-free water environment. In Experiment 3, concentration variations of water and hemolymph ammonia-N were shown in Fig. 3A. During the experiment period, the concentration of water and hemolymph ammonia-N continuously increased in AE1, AE2, and AE3, while the concentration of water ammonia-N in AEC1, AEC2, and AEC3 remained relatively stable.

In AE1, AE2, and AE3, average concentration of hemolymph ammonia-N was 1.61 mg·L^−1^ at 0 h and remained relatively stable from 0 to 24 h, while the average concentration of water ammonia-N went up steadily from 0 to 36 h and exceeded the concentration of hemolymph ammonia-N at 12 h, which indicated that shrimp sustained the concentration of hemolymph ammonia-N through excreting extra ammonia-N into the surrounding water. However, a drastic increase was observed in hemolymph ammonia-N from 24 to 72 h. At 72 h, the concentration of hemolymph ammonia-N in AE1 and AE3 was higher than the concentration of water ammonia-N, while it was at the same level in AE2. Moreover, the increase in concentration of water ammonia-N in AE1, AE2, and AE3 slightly slowed down from 36 to 72 h (Fig. 3A; Supplementary Table S8). These results together indicated that increasing concentration of water ammonia-N might hinder shrimp ammonia-N excretion and therefore lead to the accumulation of hemolymph ammonia-N, which might further imply potential alternations in organs related to ammonia-N excretion.

In Experiment 4, the concentration of water ammonia-N was measured, and the ammonia-N excretion rate was calculated for each replicate (ae1-ae45, *n* = 1). Linear regression analysis revealed a positive correlation between the concentration of water ammonia-N and exposure time (Fig. 3B; Supplementary Table S9). The ammonia-N excretion rate at 36 h-144 h was significantly lower than that at 0 h-36 h, which was in line with the turning point of concentration increasing rate of water ammonia-N concentration in Experiment 3. Although there were fluctuations in ammonia-N excretion rate among groups, the ammonia-N excretion rate decreased as water ammonia-N went up (Fig. 3C; Supplementary Table S10), which indicated the inhibition of shrimp ammonia-N excretion ability. Together with the data in Experiment 1–3, these results demonstrated that water ammonia-N suppressed shrimp ammonia-N excretion capacity and led to ammonia-N accumulation in shrimp hemolymph, which is correlated with the concentration of water ammonia-N and exposure time.

### Histopathological variation in shrimp hepatopancreas when exposed to different concentrations of water ammonia-N

**Fig. 4:**
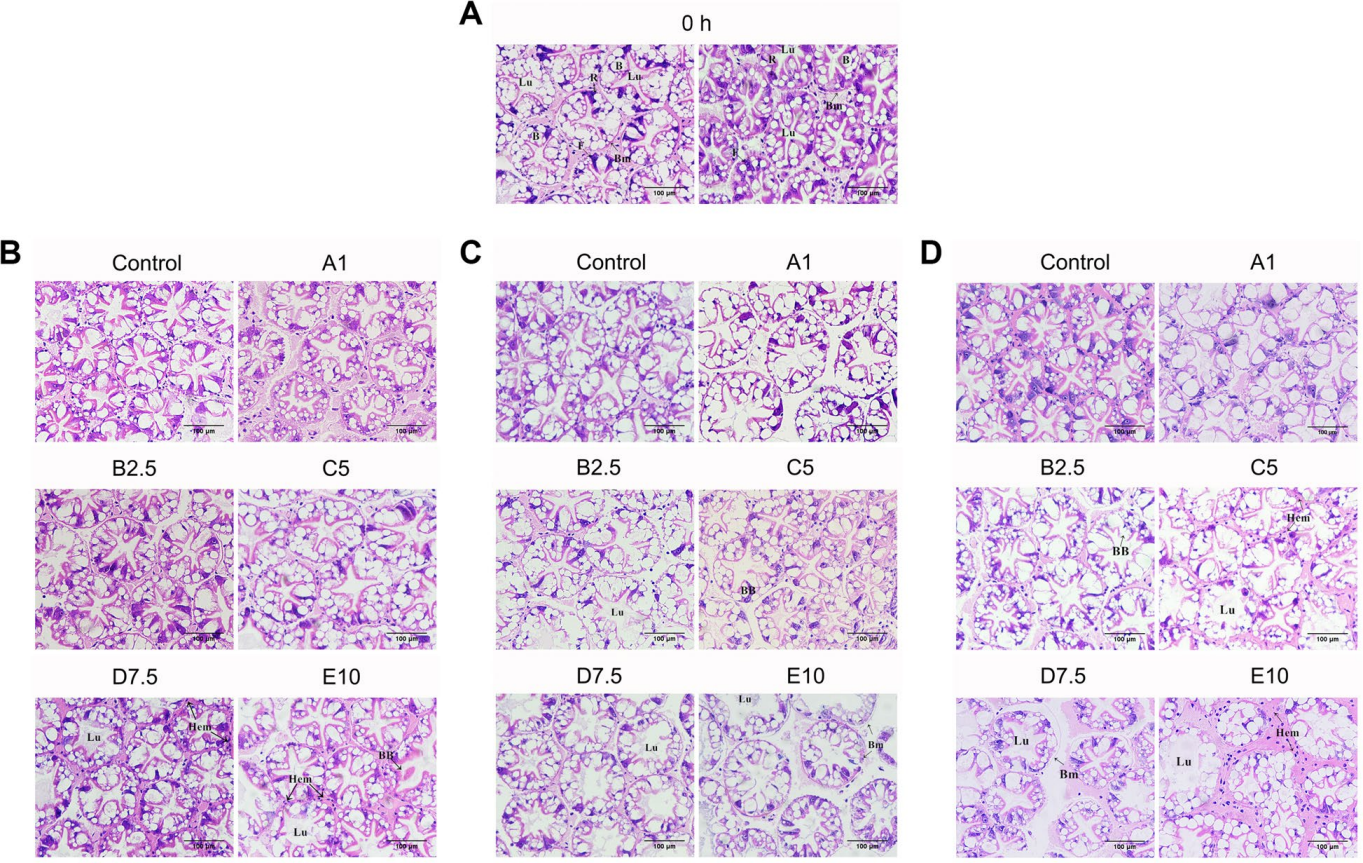
The hepatopancreas sections of shrimps exposed to different water ammonia-N concentration. Cross section of shrimp hepatopancreas tubule at 0 h (**A**), 24 h (**B**), 48 h (**C**), and 72 h (**D**). In (**B**), (**C**) and (**D**), sections of Control, A1, B2.5, C5, D7.5, and E10 were shown. Lu: Tubule lumen; B: B cell; R: R cell; F: F cell; Bm: Basement membrane; BB: Brush border; Hem: Hemocyte. Scale bar: 100 μm

Considering that hepatopancreas is crucial in regulating ammonia excretion and detoxification in aquatic animals, shrimp hepatopancreas samples were collected in Experiment 1 and were processed into slices subsequently. Histological lesions were observed in group C5, D7.5, and E10 at 24 h, 48 h, and 72 h when compared with 0 h, while hepatopancreas tissue remained intact in Control and was slightly swollen in A1 at 72 h (Fig. 4). After being exposed to ammonia-N for 72 h, the stellate lumen and the transport vesicles of B cells in A1 were slightly swollen. Rupture of the brush border of the tubule lumen was displayed in B2.5 and C5, and hemolymph cell infiltration was observed in C5. In D7.5, tubule structure was significantly disturbed, and basal membranes were dissociated, with obscured borderlines between cells and hemolymph cell infiltration. And in E10, tubule degeneration and hemolymph cell infiltration become extremely severe. Besides, at 24 h, although extensive tissue destruction and hemolymph cells infiltration were observed in D7.5 and E10, hepatopancreas cells in other groups remained intact even until 48 h. These results indicate that water ammonia-N caused damage to shrimp hepatopancreas cells, and the severity was associated with both the concentration of environmental ammonia-N and exposure time.

### Intestinal microbiota composition variation when exposed to different concentrations of water ammonia-N

**Fig. 5:**
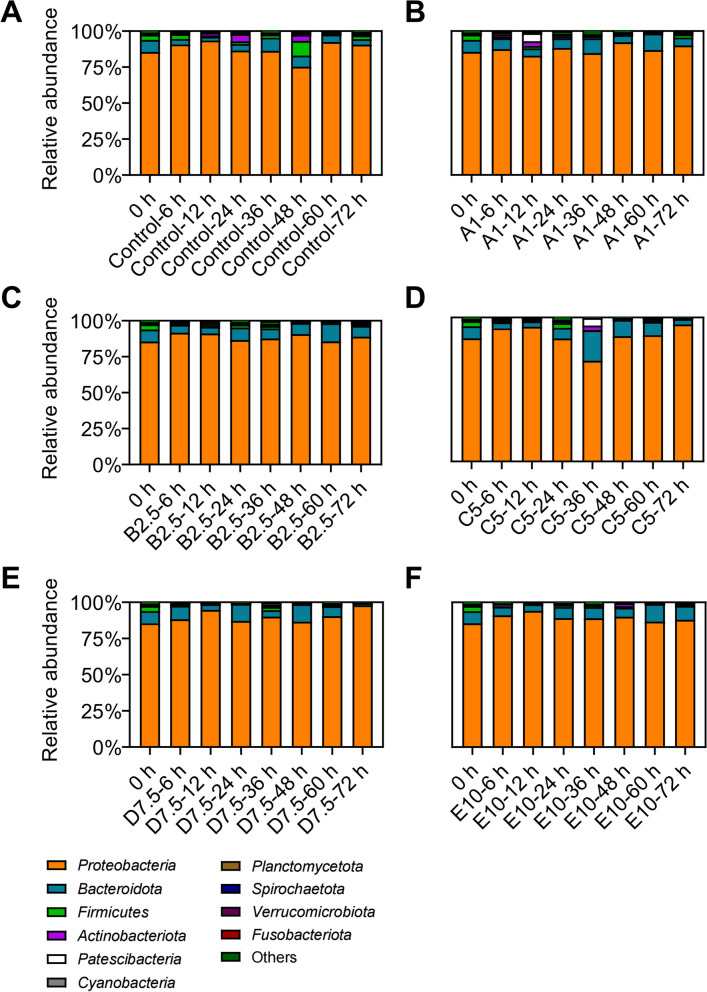
Community distribution at phylum level of intestine samples. Relative abundance of dominant phylum in Control (**A**), A1 (**B**), B2.5 (**C**), C5 (**D**), D7.5 (**E**), and E10 (**F**) were shown

A total of 6,429,932 high quality sequences were obtained from 136 shrimp intestine samples in Experiment 1, with an average of 47,279 ± 5,250 (mean ± SD) sequences per sample and the range being 32,314-69,491 sequences. After clustering sequences at 97% similarity and removing singleton, an average of 307 ± 93 bacterial OTUs were detected for each sample and classified into 42 phyla and 667 genera (Supplementary Table S11). Different bacterial composition was observed at both phylum and genus level among Control, A1, B2.5, C5, D7.5, and E10. At the phylum level, the dominant phylum was Proteobacteria, with the highest abundance of 70.00-97.93% across samples, while the following phyla were Bacteroidetes (1.51-21.11%), Firmicutes (0.20-10.09%), Actinobacteria (0.22-5.06%) and Patescibacteria (0.01-5.67%) (Fig. 5; Supplementary Fig. S2). At the genus level, *Vibrio* (0.61-98.56%), *Stenotrophomonas* (0.04-47.03%), *Shewanella* (0.25-73.91%) and *Pseudomonas* (0.003-56.85%) were the dominant genera (Supplementary Figs. S3 and S4). From 24 to 60 h, the abundance of *Achromobacter*, *Burkholderia-Caballeronia-Paraburkholderia*, and *Stenotrophomonas* decreased while the abundance of *Pseudomonas* increased. And at 48 h, as the water ammonia-N concentration went up, the abundance of *Burkholderia-Caballeronia-Paraburkholderia* decreased and reached the lowest abundance in D7.5 (Supplementary Figure S5). Despite variation in each bacterial genus observed among samples, at each taxonomic level, no significant difference in abundance was detected among samples. Also, no significant difference was detected in community alpha-diversity among different groups and sampling times, which was supported by the Chao1 index and Shannon index (*P* = 0.0647, *P* = 0.2749, ANOVA) (Table 2). Furthermore, at 48 h, 60 h, and 72 h, no significant difference was found in the abundance of *Achromobacter*, *Burkholderia-Caballeronia-Paraburkholderia*, and *Stenotrophomonas* between groups at single genus level (Supplementary Table S12)*.*

**Table 2:** Alpha-diversity across samples

	Group	6 h	12 h	24 h	36 h	48 h	60 h	72 h
Shannon index	0 h	2.92	2.92	2.92	2.92	2.92	2.92	2.92
	control	2.57	2.93	3.28	3.31	3.33	2.29	3.16
	A1	3.14	3.15	3.26	2.99	2.85	3.21	2.93
	B2.5	2.72	3.03	3.04	3.31	2.68	3.14	3.37
	C5	3.15	2.50	3.37	1.98	2.82	3.28	2.15
	D7.5	2.80	2.94	2.11	3.05	3.14	2.72	2.17
	E10	2.80	2.70	2.89	3.09	2.87	2.57	2.99
Chao1 index	0 h	274	274	274	274	274	274	274
	control	293	330	343	318	411	369	282
	A1	299	361	311	340	310	315	285
	B2.5	354	338	323	380	326	303	388
	C5	326	325	327	316	328	368	292
	D7.5	324	276	241	339	283	319	210
	E10	268	262	306	364	294	320	375

**Fig. 6:**
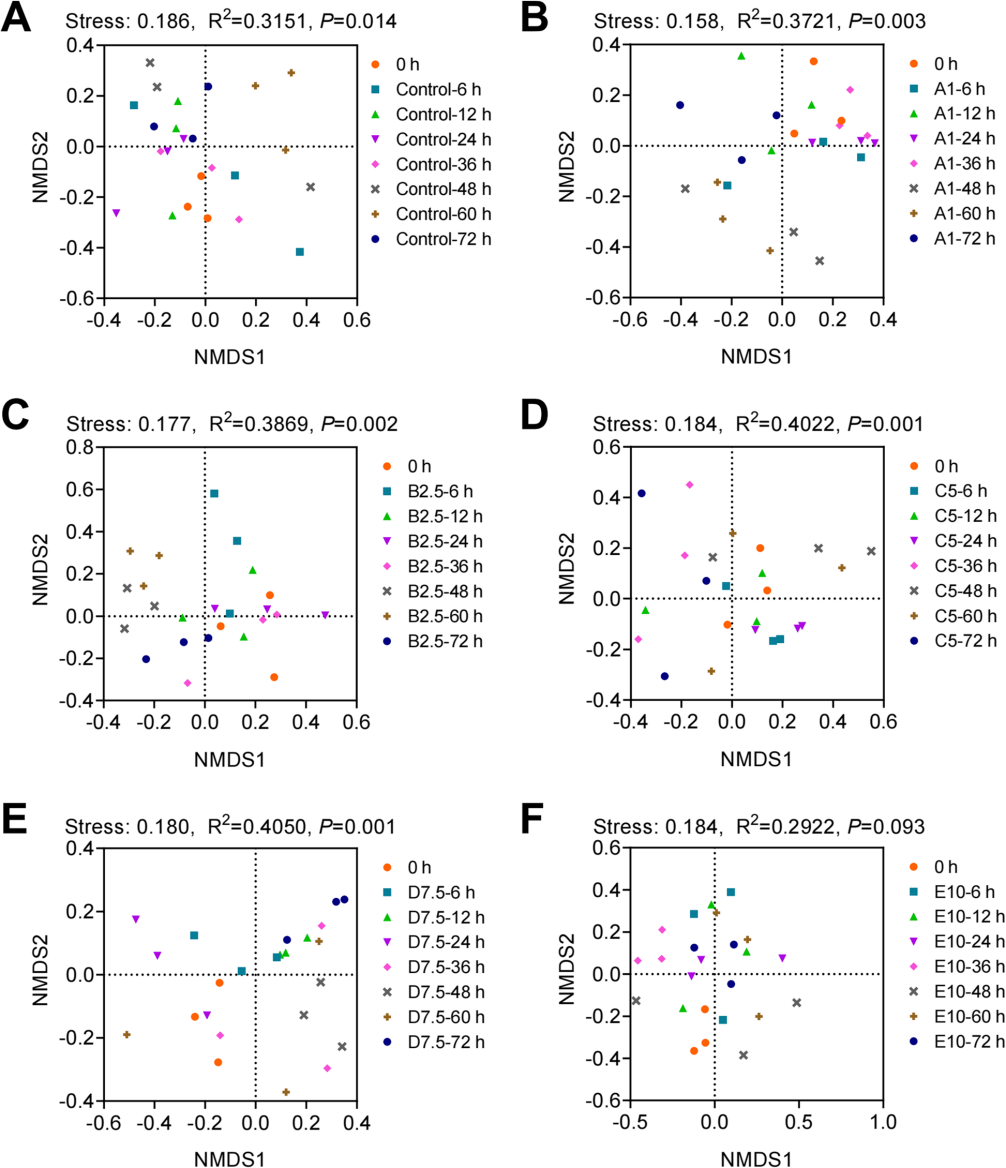
Non-metric multidimensional scaling (NMDS) analysis of the microbial community structure. Based on the Bray-Curtis distance, intestinal microbial β-diversity over time in Control (**A**), A1 (**B**), B2.5 (**C**), C5 (**D**), D7.5 (**E**), and E10 (**F**) were illustrated by NMDS analysis. The significance was determined using PERMANOVA, resulting in *R*^2^ and *p* values

NMDS analyses were performed for time series samples in different groups based on the Bray–Curtis distance (Fig. 6). In group Control, A1, B2.5, C5, and D7.5, samples collected at the same time were clustered together (Control: *P* < 0.05, A1:* P* < 0.005, B2.5:* P* < 0.005, C5: *P* < 0.005, D7.5:* P* < 0.005, E10: *P* = 0.093, PERMANOVA), which indicated that the exposure time significantly alters shrimp intestinal microbial composition. Besides, NMDS analysis was performed for different groups at 72 h (Supplementary Fig. S6). The NMDS coordination did not show significant clustering according to the concentration of water ammonia-N (*P* = 0.115, PERMANOVA), and differences within groups were also considerable (R = 0.0848, *P* = 0.186000, ANOSIM). Together, these data indicated that the concentration of water ammonia-N and exposure time could alternate the composition of shrimp intestinal microbiota, but remarkable alternations correlated with the concentration of water ammonia-N or exposure time was not identified in any microbial species.

## Discussion

The effects of high environmental ammonia-N on shrimp hemolymph ammonia-N accumulation, tissue damage, and intestinal microbial composition have been researched in several studies (Duan et al. 2021; Lin and Chen 2001; Romano and Zeng 2013; Si et al. 2019). But there is a lack of sub-chronic or chronic time-series research to characterize these patterns under realistic ammonia-N concentrations in modern intensive aquaculture ecosystems. Our study investigated the concentration of water ammonia-N and shrimp hemolymph ammonia-N in intensive aquaculture systems, and subsequently evaluated the effects of different concentrations of water ammonia-N on hemolymph ammonia-N concentration, hepatopancreas tissue, and intestinal microbial composition of *L. vannamei*, which revealed the relationship between the concentration of water ammonia-N and the concentration of shrimp hemolymph ammonia-N, hepatopancreas structure, and the composition of the intestinal microbiota.

As the most toxic form of nitrogenous waste, ammonia-N has been widely estimated as a parameter of culture water quality (Ma et al. 2013; Thakur and Lin, 2003; Visscher and Duerr 1991). Under the intensive aquaculture scenario, the concentrations of ammonia-N, nitrite-N, and nitrate-N in culture water often exceed those in natural conditions (Romano and Zeng 2013). In our study, the water ammonia-N concentration of IAPs and EPs was 0.35–1.28 mg·L^−1^ and 0.10–0.39 mg·L^−1^, respectively, which is consistent with previous findings in intensive aquaculture ponds and far lower than the LC_50_ values of *L. vannamei* (the 24, 48, 72, and 96-h LC_50_ values of ammonia-N were 59.72, 40.58, 32.15, 24.39 mg·L^−1^ at 15‰; 66.38, 48.83, 43.17, 35.4 mg·L^−1^ at 25‰; 68.75, 53.84, 44.93, 39.54 mg·L^−1^ at 35‰, respectively) (Lin and Chen 2001; Ma et al. 2013). Compared with EPs, the concentration of ammonia-N was also higher in IAPs, which might be due to the sediment ecosystem in EPs facilitating a robust nitrogen transformation process. However, the concentrations of nitrite-N and nitrate–N in our study were relatively higher in EPs compared to IAPs, which contradicts to earlier findings that concentration of nitrite-N and nitrate-N were higher in cement-bottom tanks compared to mud-bottom tanks (Ma et al. 2013). It is possible that the differences in sediment micro-ecosystem composition and function determine the concentrations of ammonia-N, nitrite-N, and nitrate-N in different ecosystems (Hou et al. 2018b; Wei et al. 2021). Therefore, the nitrogen circulation in the sediment of shrimp culture ponds might play a vital role in reducing environmental ammonia-N level and further work is required to decipher the underlying mechanisms. In addition, a significantly higher ammonia-N levels in shrimp hemolymph was observed in both EPs and IAPs in the current study, which might indicate a decline in shrimp ammonia-N excretion rate or an increase in ammonia-N influx. Therefore, though the concentration of water ammonia-N was at relatively low levels, ammonia-N still has remarkable effects on shrimp.

Various experiments in shrimp have shown significant alternation or disruption in immunological responses, osmoregulation, and histopathology when exposed to elevated ammonia-N levels (Chang et al. 2015; Dongwei Hou et al. 2018a, b; Magoč and Salzberg 2011; Si et al. 2019; Zhang et al. 2021). In the current study, increasing concentration of hemolymph ammonia-N was observed over time and peaked at 72 h, and positively correlated with the concentration of water ammonia-N in Experiment 1 (Fig. 2A and B), indicating that ammonia-N accumulation in hemolymph was determined by both the concentration of water ammonia-N and the exposure duration. Similar effects were observed in a previous study which applied a gradient of 0, 2, 10, and 20 mg·L^−1^ for water ammonia-N (Si et al. 2019). However, due to an extended exposure time, the concentration of hemolymph ammonia-N in the current study ultimately exceeded the water ammonia-N concentration, implying a dysregulation in the ammonia-N balance, which is also shown in shrimp of IAPs and EPs. It is reported that when exposed to elevated water ammonia-N, genes regulating ammonia-N excretion were significantly activated, whereas genes regulating the influx of ammonia were suppressed in *L. vannamei* (Si et al. 2019). Therefore, in Experiment 3 and 4, an elevation in ammonia-N outflow might explain the drop in ammonia-N excretion rate and the stable increase in water ammonia-N concentration, which might be achieved by facilitating active transport and vesicle transport while suppressing passive diffusion (Fig. 3A, B and C). Since the gradients of ions are essential for ammonia-N transportation, elevated environmental ammonia-N might disrupt the gradients of NH_4_^+^ and NH_3_ and thus hinder ammonia-N regulation (Romano and Zeng 2013). The toxicity and physiological condition of shrimp when exposed to ammonia-N are generally determined by the concentration of hemolymph ammonia-N. In Experiment 2, we found that pristine water alleviated the effects of elevated water ammonia-N on shrimp by reducing the concentration of hemolymph ammonia-N and mortality, though the mortality rate in group c50 and Tc50 ultimately reached 100%. These phenomena suggest that the recovery effect might hinge on the duration and concentration of water ammonia-N in the previous exposure. Romano and Zeng (2010) found that the hemolymph ammonia-N concentration and the gill structure were totally recovered after being exposed to sub-lethal water ammonia-N levels and then transferred to pristine water for 96 h in *Portunus pelagicus*. Differences in species characteristics (e.g., LC_50_) and the recovery duration could explain the discrepancies between studies. However, changes in ammonia-N regulation strategies following elevated ammonia-N exposure are highly species-specific. Therefore, to thoroughly characterize the impacts on shrimp ammonia-N regulation, additional research integrating histopathology is imperative to precisely investigate the structure variation in gills during the exposure and recovery phase.

The hepatopancreas is crucial in energy metabolism and immune defense of *L. vannamei*, and an indicator of structural and functional alternation in immunopathology studies (Wang et al. 2021; Zhao et al. 2017)*.* Hepatopancreas structure under water ammonia-N stress demonstrated that the severity of tissue damage heavily depends on the concentration of water ammonia-N and exposure time (Fig. 4). The consistency between hepatopancreas structure and the concentration of hemolymph ammonia-N hints that ammonia-N in the circulatory system plays an important role in regulating the functions of different organs. Similar impairments were also identified in the intestine barrier of *L. vannamei* and gills of *P. pelagicus* under elevated water ammonia-N (Duan et al. 2018; Romano and Zeng 2010). The extensive tubule destruction and hemolymph cell infiltration imply possible functional changes or even loss in hepatopancreas cells. Wang et al. (2021) has confirmed that cellular energy levels were downregulated by ammonia-N stress, which induced increased lipid decomposition and rendered higher energy levels in hepatopancreas cells. Besides, in hepatopancreas, immune defense inhibition generated by differentially expressed genes under ammonia-N stress was observed through transcriptome sequencing (Zhang et al. 2021). These results together demonstrate how ammonia-N systematically disrupts the homeostasis in hepatopancreas and predisposes shrimp to pathogen invasion. It was proven that in the gills of *P. pelagicus*, structure variation caused by elevated water ammonia-N recovered after being treated with pristine water (Romano and Zeng 2010). However, whether transferring *L. vannamei* to pristine water could recover the hepatopancreatic destruction caused by ammonia-N is unclear.

The intestinal microbiota and gastrointestinal tract are considered to lie at the intersection of the internal and external environment (Hou et al. 2020; Sullam et al. 2012). Changes in environmental factors directly shape the intestinal microbiota and metabolic processes (Duan et al. 2018; Qi et al. 2017; Wei et al. 2021). As multifactorial pathogenesis has been proposed and substantiated in human and animal diseases, the intestinal microbiota has received a lot of attention in the research of disease occurrence and progression for its ability to reflect the health condition of the host (Gilbert et al. 2016; Metwaly et al. 2022). Duan et al. (2021) has demonstrated that the composition of the intestinal microbiota changed after being exposed to 20 mg·L^−1^ ammonia-N for 72 h in *L. vannamei*, with bacterial diversity decreasing. At the phylum level, the relative abundance of Proteobacteria and Planctomyetes decreased while the relative abundance of Bacteroidetes increased. At the genus level, the relative abundance of *Formosa*, *Ruegeria*, *Nautella*, and *Pseudoalteromonas* increased while the relative abundance of *Photobacterium*, *Rhodopirellula*, and *Planctomyces* was reduced (Duan et al. 2018). In contrast, an opposite trend was found in another study: at the phylum level, the relative abundance of Proteobacteria and Firmicutes increased while the relative abundance of Bacteroidetes decreased. At the genus level, the relative abundance of *Bacteroides* was increased while the relative abundance of *Formosa* and *Ruegeria* was decreased when exposing *L. vannamei* to 15 mg·L^−1^ ammonia-N for 72 h (Duan et al. 2021). Moreover, a decrease in the relative abundance of Bacteroidetes was also found in an experiment in which *L. vannamei* was exposed to 15 mg·L^−1^ ammonia-N for 96 h (Lv et al. 2021). In the present study, different concentrations of water ammonia-N and exposure time both altered the intestinal microbial composition. Consistent with earlier findings, the dominant phyla were Proteobacteria, Bacteroidetes, and Firmicutes, and the dominant genus was *Vibrio.* Although the composition of the microbiota altered, there were no significant differences among groups in either concentration of ammonia-N or exposure time. However, the contrary trend in species abundance and the insignificant changes between groups reflect the complexity of the ammonia-N regulatory mechanism in the intestine. Nevertheless, in the current study, at the genus level, the variation in relative abundance of *Achromobacter*, *Burkholderia-Caballeronia-Paraburkholderia*, *Stenotrophomonas*, and *Pseudomonas*, which are both related to human infectious diseases and bring about negative effects on the host, showing similar pattern when exposed to different concentrations of water ammonia-N (Supplementary Figs. S4 and S5) (Hossain, 2014; Lara-Oya, 2022). In view of the high relative abundance of these species across samples (Supplementary Fig. 1A), the increase in exposure time might exacerbate host health condition by facilitating pathogen invasion and suppressing host immune defense. Therefore, it is necessary to better understand the variation in microbial function and how bacteria interact with each other and the host when exposed to different concentrations of ammonia-N.

In summary, the concentrations of ammonia-N, nitrite-N, and nitrate-N in water and shrimp hemolymph of the intensive aquaculture ecosystem were evaluated in IAPs and EPs, and four experiments were designed in laboratory condition to explore the effects of different concentrations of water ammonia-N and exposure time on hemolymph, hepatopancreas, and the intestinal microbiota of *L. vannamei*. The overall results provide evidence for understanding the effects of different concentrations and exposure time of water ammonia-N on shrimp hemolymph ammonia-N level, hepatopancreas structure, and intestinal microbiota, and thus provide a theoretical basis for scientific culture management and microecological regulation in the shrimp industry.

## Supplementary Information

**Additional file 1:** **Supplementary Table S1. **Ammonia-N, nitrite-N, and nitrate-N concentration in water and shrimp hemolymph across sampling sites.** Supplementary Table S2. **Comparison of Ammonia-N, nitrite-N, and nitrate-N concentration between water and shrimp hemolymph in different aquaculture ponds.** Supplementary Table S3. **Difference analysis of hemolymph ammonia-N concentration of different groups in Experiment 1.** Supplementary Table S4. **Hemolymph ammonia-N concentration of different groups over time in Experiment 1.** Supplementary Table S5. **Mortality of shrimp in different groups over time in Experiment 2.** Supplementary Table S6. **Variation of hemolymph ammonia-N of different groups over time in Experiment 2.** Supplementary Table S7. **Difference analysis of hemolymph ammonia-N concentration between untreated and transfer groups in Experiment 2.** Supplementary Table S8. **Variation of water and hemolymph ammonia-N concentration over time in Experiment 3. **Supplementary Table S9. **Variation of water ammonia-N concentration over time in Experiment 4.** Supplementary Table S10. **Ammonia-N excretion rate of *Litopenaeus vannamei* at different periods during ammonia excretion process in Experiment 4.** Supplementary Table S11. **16S rRNA sequencing information.** Supplementary Table S12. **Difference analysis of relative abundance of *Achromobacter*, *Burkholderia*-*Caballeronia*-*Paraburkholderia*, and *Stenotrophomonas* at 48 h, 60 h, and 72 h.

**Additional file 2:** **Supplementary Fig. S1. **Design of Experiment 1-4. **Supplementary Fig. S2. **Community distribution at phylum level of intestine samples at 72 h. **Supplementary Fig. S3. **Heatmap of bacterial distribution across samples at genus level.Rows represent the 35 most abundant genera. The log10-transformed relative percentage of each genus is depicted by color intensity. **Supplementary Fig. S4.** Heatmap of bacterial distribution across samples at genus level. **Supplementary Fig. S5.** Top 10 most abundant genera at 48 and 72 h. **Supplementary Fig. S6.** NMDS analysis of samples of different water ammonia-N concentration at 72 h.

## Data Availability

The 16S rRNA gene sequencing data used in this study are available in the NCBI Short Read Archive (https://www.ncbi.nlm.nih.gov/sra) under Bioproject PRJNA877447.
