# Supplementary material for: Effects of water ammonia nitrogen on hemolymph and intestinal microbiota of Litopenaeus vannamei
Source: Adv Biotechnol (Singap). 2024 Jan 26;2(1):1. doi: 10.1007/s44307-023-00008-2 (PMC11740837; doi:10.1007/s44307-023-00008-2)
Supplement: Supplementary file 2 — Additional file 2: Supplementary Fig. S1. Design of Experiment 1-4. Supplementary Fig. S2. Community distribution at phylum level of intestine samples at 72 h. Supplementary Fig. S3. Heatmap of bacterial distribution across samples at genus level.Rows represent the 35 most abundant genera. The log10-transformed relative percentage of each genus is depicted by color intensity. Supplementary Fig. S4. Heatmap of bacterial distribution across samples at genus level. Supplementary Fig. S5. Top 10 most abundant genera at 48 and 72 h. Supplementary Fig. S6. NMDS analysis of samples of different water ammonia-N concentration at 72 h. [file 44307_2023_8_MOESM2_ESM.docx]

Supplementary Figures for

**Effects of Water Ammonia Nitrogen on Hemolymph Ammonia Nitrogen, Hepatopancreas Histopathology and Intestinal Bacterial Communities in *Litopenaeus vannamei***

Xuanting Li^1,†^, Xisha Deng^1,†^, Dongwei Hou^1^, Shenzheng Zeng^1^, Zhixuan Deng^1^, Renjun Zhou^1^, Lingyu Zhang^2^, Qilu Hou^1^, Qi Chen^1^, Shaoping Weng^1,2,3^, Jianguo He^1,2,3*^, Zhijian Huang^1,3*^

^1^State Key Laboratory of Biocontrol, Southern Marine Sciences and Engineering Guangdong Laboratory (Zhuhai), School of Marine Sciences, Sun Yat-sen University, Guangzhou 510275, P. R. China

^2^School of Life Sciences, Sun Yat-sen University, Guangzhou 510275, P. R. China

^3^Maoming Branch, Guangdong Laboratory for Lingnan Modern Agricultural Science and Technology, Maoming 525435, P. R. China

*Corresponding Authors
Jianguo He, lsshjg@mail.sysu.edu.cn

Zhijian Huang, [lsshzhj@mail.sysu.edu.cn](mailto:lsshzhj@mail.sysu.edu.cn)

This supplementary file contains the following:
Supplementary Figure S1-S6


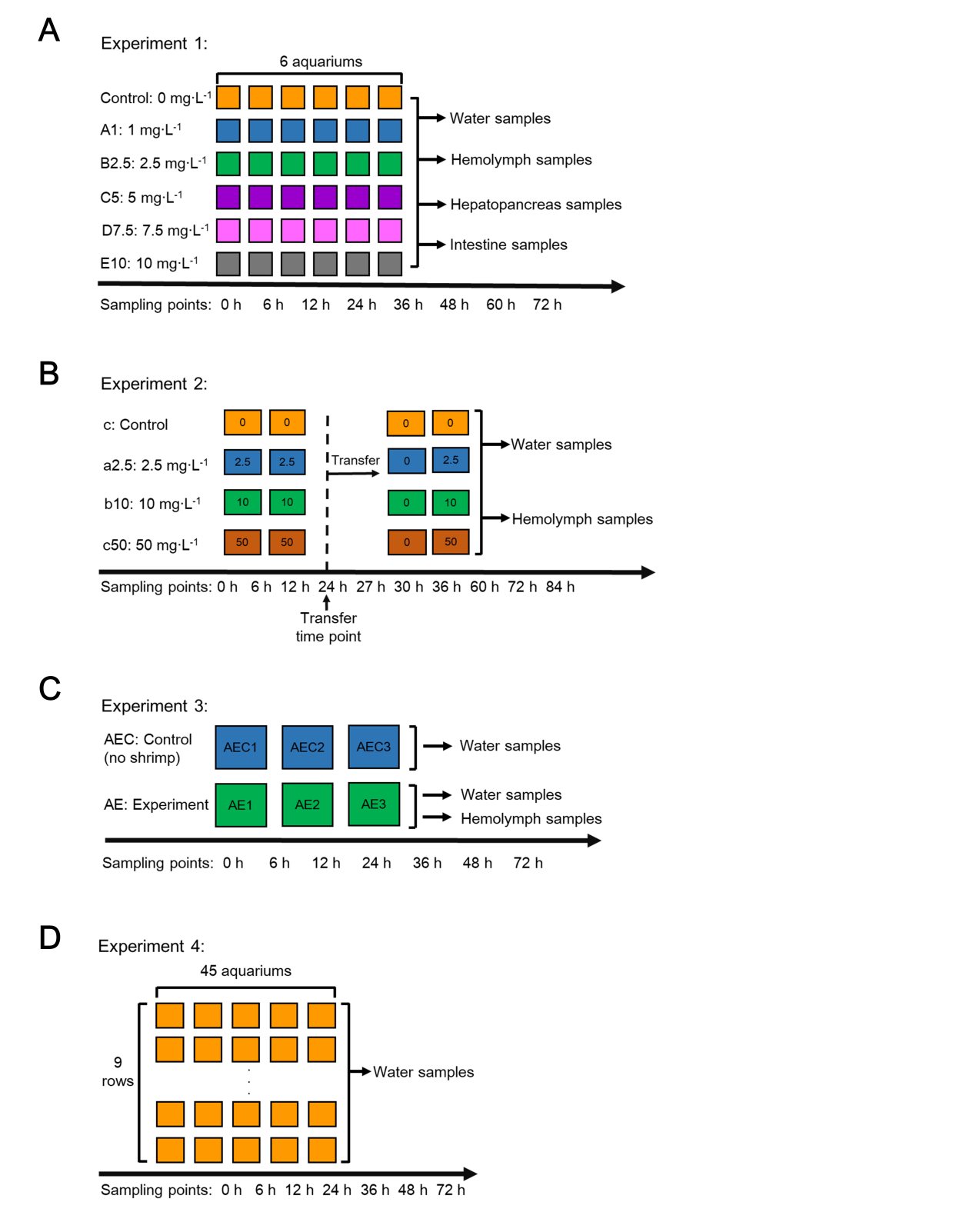


**Supplementary Fig. S1 Design of Experiment 1-4.** (A) Experiment 1. (B) Experiment 2. Numbers in the boxes represent the concentration of water ammonia-N in each aquarium. (C) Experiment 3. (D) Experiment 4


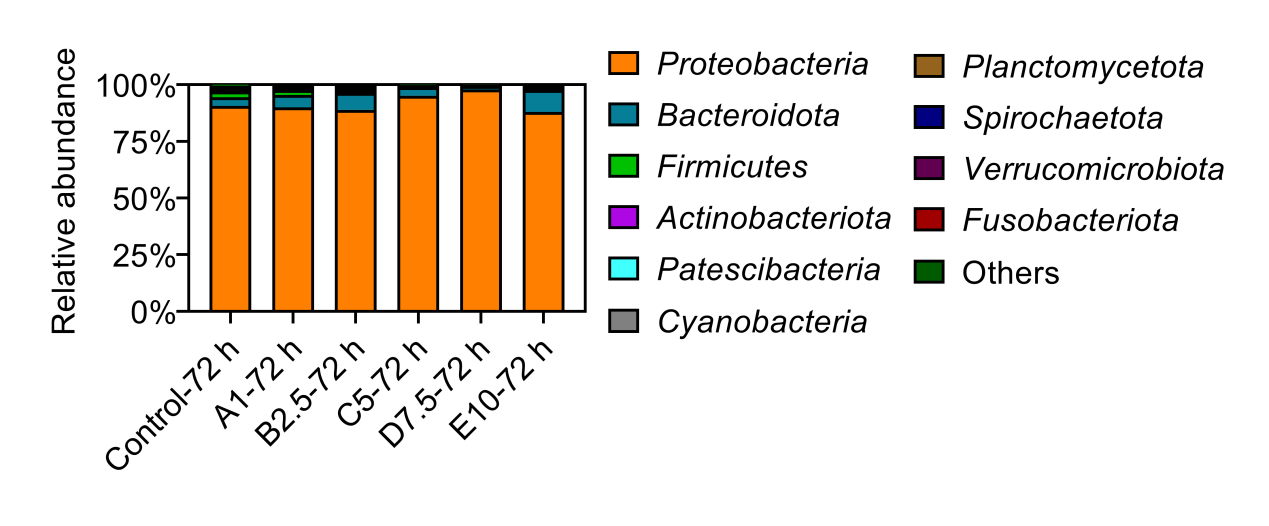


**Supplementary Fig. S2 Community distribution at phylum level of intestine samples at 72 h.** Relative abundance of dominant phylum of different water ammonia-N concentration at 72 h


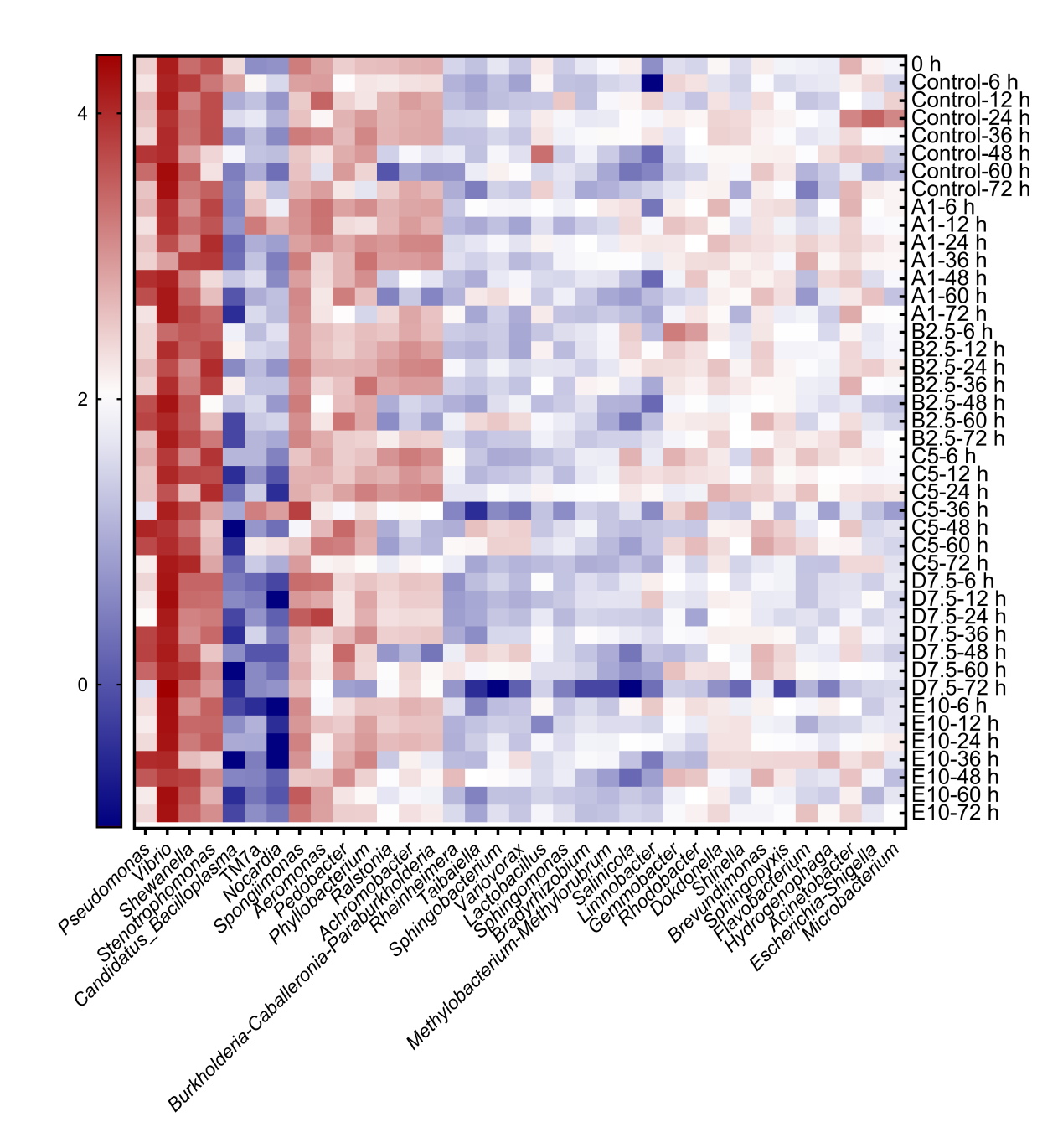


**Supplementary Fig. S3 Heatmap of bacterial distribution across samples at genus level.** Rows represent the 35 most abundant genera. The log10-transformed relative percentage of each genus is depicted by color intensity


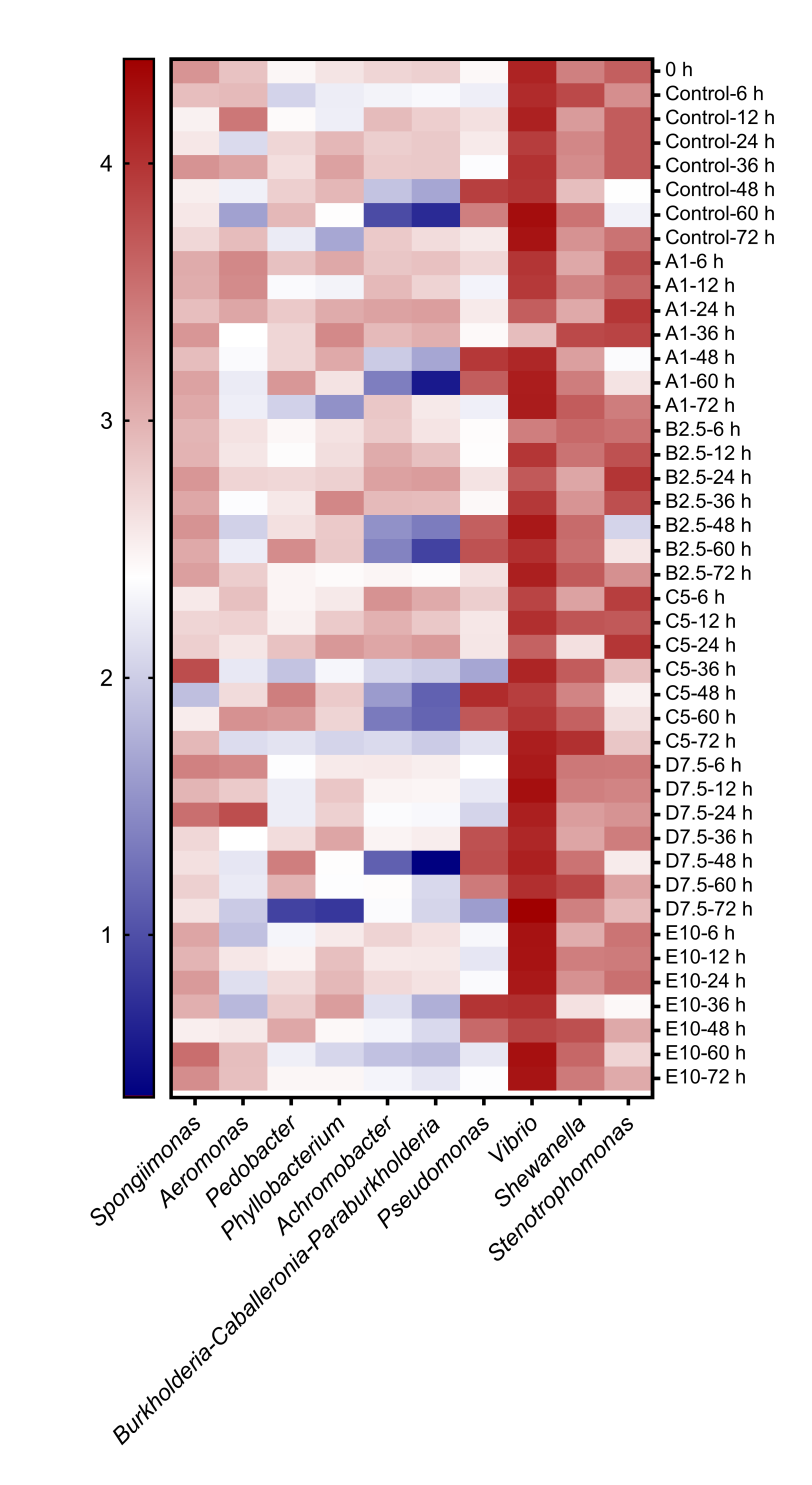


**Supplementary Fig. S4 Heatmap of bacterial distribution across samples at genus level.** Rows represent the 10 most abundant genera. The log10-transformed relative percentage of each genus is depicted by color intensity


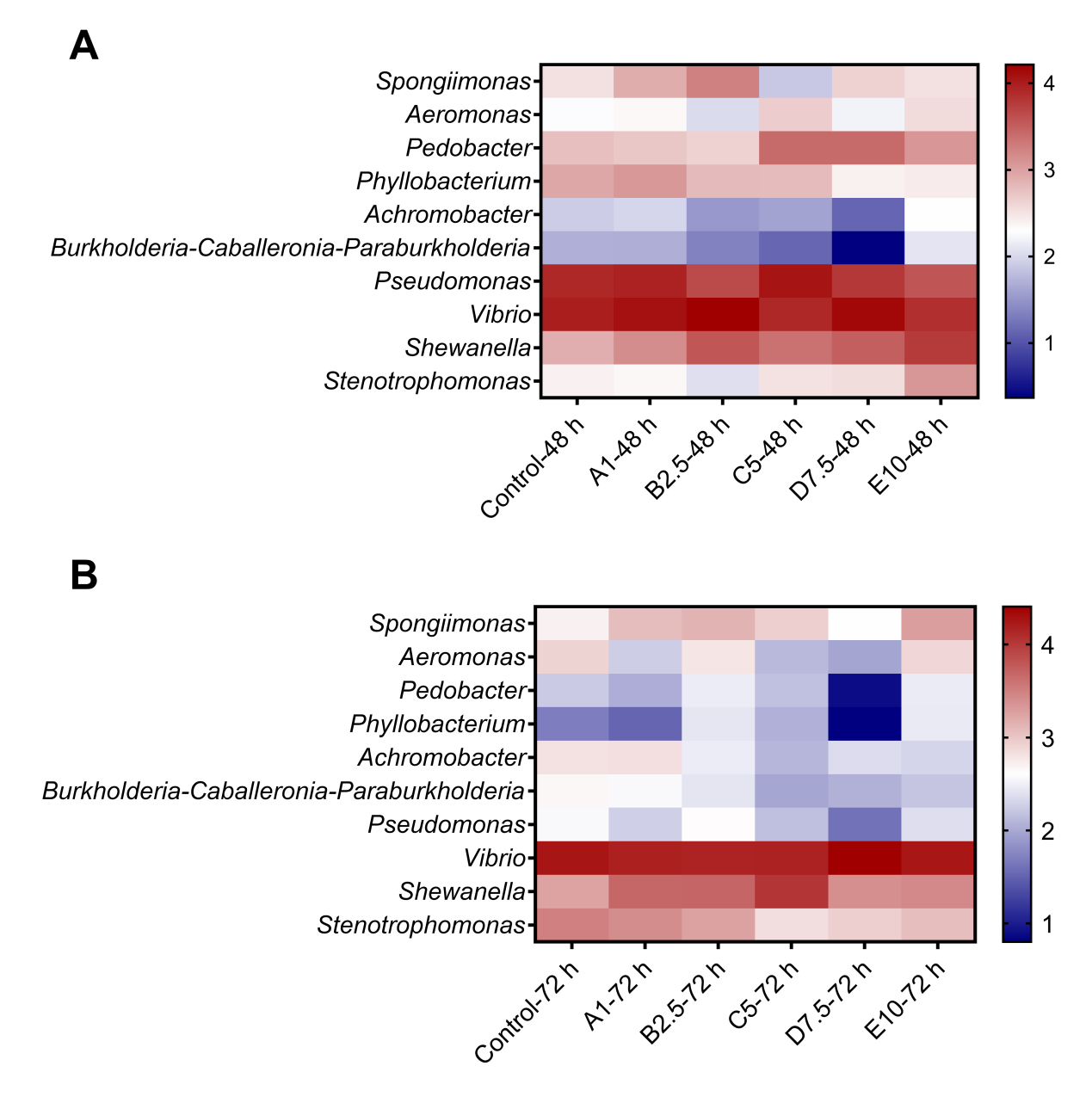


**Supplementary Fig. S5** **Top 10 most abundant genera at 48 and 72 h.** (A) Heatmap of bacterial distribution in intestine samples with different water ammonia-N concentration at 48 h. (B) Heatmap of bacterial distribution in intestine samples with different water ammonia-N concentration at 72 h. Rows represent the 10 most abundant genera. The log10-transformed relative percentage of each genus is depicted by color intensity


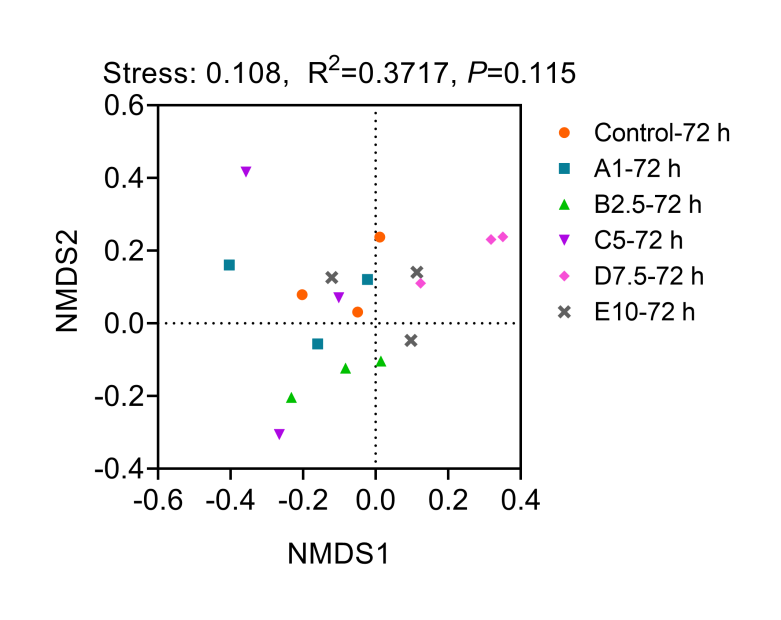


**Supplementary Fig. S6** NMDS analysis of samples of different water ammonia-N concentration at 72h. The significance was determined using PERMANOVA, resulting in R^2^ and *p* values
